# Psychometric evaluation of the Chinese modified perceived neighborhood cohesion scale in older adults at high risk of cognitive impairment

**DOI:** 10.1002/alz.088249

**Published:** 2025-01-09

**Authors:** Yi‐Chen Chiu, Mei‐Hsuan Lin, Pei‐Chen Hsu, Wen‐Chuin Hsu, Yi‐Chun Chen

**Affiliations:** ^1^ School of Nursing, College of Medicine, Healthy Aging Research Center, Chang Gung University, Taoyuan Taiwan; ^2^ School of Nursing, College of Medicine, Chang Gung University, Taoyuan Taiwan; ^3^ Chang Gung Memorial Hospital and Dementia Center, Taoyuan Taiwan; ^4^ Chung Gung University, Taoyuan Taiwan; ^5^ Chang Gung Memorial Hospital, Taoyuan Taiwan

## Abstract

**Background:**

The global increase in dementia cases underscores the need for community‐based services, particularly for older adults who spend significant time in their neighborhoods. This context profoundly influences cognitive function and quality of life, especially in those at high risk of cognitive impairment. Despite its importance, neighborhood cohesion has not been extensively studied in this group. This study aimed to assess the reliability and validity of the Chinese Modified Perceived Neighborhood Cohesion Scale (CMPNCS) in Northern Taiwanese older adults at high risk of cognitive impairment.

**Method:**

A cross‐sectional, mixed‐methods approach was employed. Qualitative interviews with 10 stakeholders, including at‐risk older adults and community leaders in Northern Taiwan, informed the adaptation of the English Perceived Neighborhood Cohesion Scale into a 13‐item Chinese version. Quantitative phase applied the convenient sampling method to recruit 125 older adults with high risk of cognitive impairment in communities in Northern Taiwan. A trained research assistant administered the revised scale face‐to‐face to 125 older adults identified as high‐risk for cognitive impairment. Psychometric properties assessed included expert validity (Content Validity Index), exploratory factor analysis, internal consistency (Cronbach’s Alpha), and 7‐day test‐retest and inter‐rater reliability.

**Results:**

The quantitative phase results are listed as follows. The participant demographic was 31.2% male and 68.8% female, primarily aged 70‐74 (27.2%) and ≥75 (27.2%). The Content Validity Index was 0.94. Exploratory factor analysis yielded a three‐factor solution: Trust in the Neighborhood (Cronbach’s Alpha = 0.84), Community Leader Action (Cronbach’s Alpha = 0.68), and Cohesion in the Neighborhood (Cronbach’s Alpha = 0.77). Overall internal consistency was high (Cronbach’s Alpha = 0.88), with excellent 7‐day test‐retest (0.92) and inter‐rater reliability (0.99).

**Conclusion:**

The CMPNCS exhibits strong psychometric properties, making it a valuable tool in nursing research. It enables the assessment of neighborhood cohesion and the exploration of its relationship with cognitive function in at‐risk older adults. The findings can guide community health nurses in enhancing perceived community cohesion and developing tailored environmental interventions for this population.

